# Gut microbiota alterations in patients with non‐small‐cell lung cancer undergoing chemoradiotherapy

**DOI:** 10.1002/1878-0261.70326

**Published:** 2026-09-07

**Authors:** Hanne Marte Nymoen, Zhi Zhao, Henrik Horndalsveen, Maria Moksnes Bjaanæs, Bjørn Henning Grønberg, Tarje Halvorsen, Tesfaye Madebo, Marianne Aanerud, Jussi Koivunen, Kersti Oselin, Saulius Cicenas, Nina Helbekkmo, Jarkko Ahvonen, Maria Silvoniemi, Tine Norman Alver, Tonje Dalen, Åsa Kristina Öjlert, Saima Jamil Farooqi, Åslaug Helland, Vilde Drageset Haakensen

**Affiliations:** ^1^ Department of Cancer Genetics Oslo University Hospital Norway; ^2^ Faculty of Medicine University of Oslo Norway; ^3^ Department of Oncology Oslo University Hospital Norway; ^4^ Faculty of Medicine and Health Sciences Norwegian University of Science and Technology Trondheim Norway; ^5^ Department of Oncology St Olavs Hospital Trondheim University Hospital Norway; ^6^ Department of Clinical and Molecular Medicine Norwegian University of Science and Technology Trondheim Norway; ^7^ Department of Pulmonology Stavanger University Hospital Norway; ^8^ Department of Clinical Science University of Bergen Norway; ^9^ Department of Thoracic Medicine Haukeland University Hospital Bergen Norway; ^10^ Department of Oncology and Radiotherapy Oulu University Hospital Finland; ^11^ Medical Research Center Oulu Finland; ^12^ Oncology and Haematology Clinic North Estonia Medical Centre Tallinn Estonia; ^13^ Department of Thoracic Surgery and Oncology Nacionalinis vėžio institutas Vilnius Lithuania; ^14^ Clinic of Internal Diseases, Family Medicine and Oncology Vilnius University Faculty of Medicine Lithuania; ^15^ Department of Pulmonology University Hospital of North Norway Tromsø Norway; ^16^ Department of Oncology Tampere University Hospital, Tays Cancer Center Finland; ^17^ Department of Pulmonary Medicine Turku University Central Hospital Finland

**Keywords:** biomarkers, chemoradiotherapy, microbiota diversity, Non‐small‐cell lung cancer

## Abstract

Growing evidence suggests that gut microbial features may predict—and potentially modulate—responses to cancer therapy, particularly immunotherapy. However, the impact of concurrent chemoradiotherapy (CRT) on the gut microbiota remains less well‐understood. This knowledge gap is especially relevant in locally advanced non‐small‐cell lung cancer (NSCLC), where CRT typically precedes immunotherapy. We therefore investigated whether CRT alters gut microbial composition and whether such changes are associated with survival outcomes. Fecal samples were collected at three time points: prior to CRT (baseline), at completion of CRT, and immediately before initiation of consolidation immunotherapy, from 62 patients with locally advanced NSCLC. Microbiota profiling was performed using a qPCR‐based panel (PMP™) targeting 108 prevalent microbial taxa. Within‐sample (alpha) and between‐sample (beta) bacterial diversity were assessed across time points and clinical subgroups defined by antibiotic exposure and survival outcomes. Alpha diversity remained stable from baseline to completion of CRT (all *P* > 0.60). Patients who received broad‐spectrum antibiotics during CRT had significantly lower alpha diversity compared with those who did not receive antibiotics (*P* = 0.017), reflecting a transient between‐group difference. Beta diversity differed modestly but significantly at baseline between survival groups (progression‐free survival ≥ 18 vs < 18 months; PERMANOVA *R* ^2^ = 0.028, *P* = 0.045). The gut microbiota appears largely stable during CRT for locally advanced NSCLC but is susceptible to disruption by antibiotic use. Baseline beta diversity differences between survival groups may signal a potential role for gut microbial composition as a future preliminary biomarker and warrant further investigation.

## Graphical abstract

We investigated the impact of concurrent chemoradiotherapy (CRT) on the gut microbiota in patients with locally advanced non‐small‐cell lung cancer. Overall gut microbiota composition remained largely stable throughout CRT while antibiotic exposure may influence microbial diversity. Subtle baseline differences in microbial composition across survival groups warrant further investigation into whether selected gut microbial taxa may have prognostic value. Abbreviation: IO, immuno‐oncology.

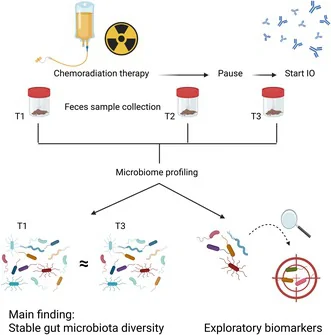

**Abbreviations**
CRT: chemoradiotherapy
CT: Computed tomography
DART: The Durvalumab After ChemoRadiotherapy study
LDA: Linear Discriminant Analysis
LEfSe: Linear Discriminant Analysis Effect Size
NSCLC: non‐small‐cell lung cancer
OS: overall survival
PCoA: principal coordinate analysis
PD‐L1: programmed death‐ligand 1
PFS: progression‐free survival
PMP: Precision Microbiome Profiling
qPCR: quantitative real‐time polymerase chain reaction
RECIST: Response Evaluation Criteria In Solid Tumors
SDI: Shannon Diversity Index

## Introduction

Despite significant advances in lung cancer treatment, the disease remains a leading cause of cancer‐related mortality worldwide [1]. Non‐small‐cell lung cancer (NSCLC) accounts for approximately 85% of all lung cancer cases [2]. For patients with locally advanced, unresectable disease, concurrent chemoradiotherapy (CRT) remains the standard of care. In Europe, patients with nonprogressive disease after CRT‐ and PD‐L1‐positive tumors are eligible for subsequent immunotherapy [3]. Immunotherapy has transformed NSCLC treatment; however, the mechanisms underlying differential tumor responses remain incompletely understood, underscoring the need for reliable predictive biomarkers.

The human microbiota, a diverse community of microorganisms including bacteria, viruses, fungi, and archaea, resides on and within the body. This vast microbial ecosystem is estimated to comprise trillions of cells and genes, roughly equal in number to human body cells [4]. These microorganisms exist in a dynamic balance, exerting neutral, beneficial, or unfavorable effects on the host, the latter described as dysbiosis. The majority of human‐associated microbes are bacteria, predominantly residing in the colon, which constitutes approximately 97% of the microbiota [5]. Research has predominantly focused on this region and its implications for human health. Numerous studies have linked gut microbiota composition to a wide spectrum of conditions, including gastrointestinal, immunological, cardiovascular, metabolic, and psychiatric disorders [6, 7].

Greater microbial diversity—defined by the variety and evenness of an ecosystem—generally correlates with improved outcomes across disease contexts [8, 9, 10], and in oncology, the most robust associations have been reported for immunotherapy response and toxicity [11, 12, 13].

The gut microbiota can modulate drug efficacy and toxicity through direct and indirect mechanisms [14, 15]. Increased abundance of *Akkermansia muciniphila* has been associated with enhanced response to doxorubicin in murine models of triple‐negative breast cancer and to FOLFOX and cisplatin [16, 17, 18], whereas *Mycoplasma hyorhinis* and certain Gammaproteobacteria species can inactivate fluoropyrimidines and gemcitabine [19, 20].

The microbiota–radiotherapy (RT) relationship is less well‐characterized, with most studies focusing on gastrointestinal RT [21, 22, 23, 24]. Preclinically, vancomycin‐induced microbiota alterations enhanced tumor response and elicited abscopal effects, and lung irradiation induced microbiota changes in both lung and gut compartments [25, 26]. Clinically, higher microbiota diversity at two weeks after initiation of CRT was associated with improved progression‐free survival (PFS) in lung cancer [27].

Given the growing body of evidence, the interplay between CRT and the gut microbiota warrants investigation in locally advanced NSCLC, where CRT precedes immunotherapy. This study explores gut microbiota dynamics during CRT in 62 patients prior to consolidation immunotherapy.

## Methods and materials

### Study design, cohort, and sample collection

Patients with unresectable locally advanced NSCLC were enrolled in the prospective DART study (ClinicalTrials.gov identifier: NCT04392505) between May 2020 and September 2023. Patients were recruited from oncology centers in Norway, Finland, Estonia, and Lithuania. Key inclusion criteria included an ECOG performance status of 0–1, age ≥ 18 years, adequate organ function, no active inflammatory disorder, and absence of active concomitant malignancy (all eligibility criteria are listed in Table S1). All patients were planned for definitive CRT, consisting of RT to the primary lung tumor and local lymph node metastases delivered to a total dose of 60–66 Gy in 2 Gy fractions, concomitantly with two cycles of cisplatin or carboplatin plus etoposide administered three weeks apart. Patients without disease progression after CRT then received durvalumab 1500 mg intravenously every 4 weeks until disease progression, unacceptable toxicity, or 12 months of treatment. Computed tomography (CT) scans of the thorax and upper abdomen were performed at baseline, following the completion of CRT, and subsequently every three months for up to two years or upon clinical suspicion of disease progression. Stool samples were collected on filter papers at three predefined time points: prior to initiation of CRT (T1), immediately following the completion of CRT (T2), and prior to initiation of immunotherapy approximately four weeks after completion of CRT (T3) (Fig. S1). Patients with nonprogressive disease after CRT who received at least one cycle of durvalumab and provided at least one stool sample at T1‐T3 were included in this study.

### DNA extraction and microbiome profiling

DNA extraction from the feces was performed at Bio‐Me (Oslo, Norway) using a commercially developed panel. From each filter card, three 6‐mm disks were punched out and placed into wells on MagMax™ 96 Deep Well Plates (Thermo Fisher Scientific, Waltham, USA). Following bead‐beating to break down microbial cell walls, DNA was extracted using the Microbiome MagMAX Ultra kit (Thermo Fisher Scientific) according to the manufacturer's instructions on the KingFisher™ Flex system (Thermo Fisher Scientific). DNA concentration was measured using the Quant‐iT™ picoGreen™ dsDNA Reagent (Thermo Fisher Scientific). The gut microbiota composition was assessed using a validated quantitative PCR (qPCR) method (Bio‐Me's Precision Microbiome Profiling [PMP™]), which uses TaqMan™ technology on the OpenArray^®^ platform (Thermo Fisher Scientific) to target 108 commonly occurring and well‐characterized taxa in the human gut. Standard curves for the qPCR assays were established using reference material quantified by fluorescence with the Quant‐iT™ PicoGreen™ dsDNA Reagent (Thermo Fisher Scientific). The absolute copy number of each target (genomic copies per μL) was interpolated from the standard curves. To normalize the data, absolute copy numbers were adjusted according to the total DNA concentration of each sample, obtaining a normalized absolute quantification (genomic copies per nanogram of purified DNA).

### Statistical analysis

Preprocessing harmonized bacterial taxa to a common taxonomic resolution, resulting in 100 species‐level taxa for downstream analyses. Relative abundances were calculated by dividing the normalized absolute quantification of each species by the total normalized absolute quantification within each sample. At each time point (T1, T2, T3), microbial richness was summarized as the median of observed taxa counts across all samples. Alpha diversity (within‐sample richness and evenness) was assessed using the Shannon Diversity Index (SDI) based on relative abundance data. Alpha diversity across time points was assessed using paired Wilcoxon signed‐rank tests. Longitudinal patterns in alpha diversity were explored using longitudinal k‐means clustering. For survival analyses, PFS and overall survival (OS) were estimated using the Kaplan–Meier method and compared using the log‐rank test. Survival time was calculated from the date of inclusion to the first documented progression (according to the Response Evaluation Criteria In Solid Tumors; RECIST) or death, respectively. The data cutoff for follow‐up was 1 August 2025; patients without progression or death were censored at that date. For analyses requiring dichotomization, patients were classified according to PFS (over or under 18 months), and the same cutoff was applied to OS. Sensitivity analyses were performed using an alternative cutoff of 12 months. The 18‐month cutoff was selected as a clinically meaningful landmark in locally advanced NSCLC, reflecting a follow‐up interval during which disease progression is expected to occur in a substantial proportion of patients. This time point also aligns with previously reported PFS landmarks in studies evaluating CRT and immunotherapy in advanced NSCLC [3]. For survival analyses involving alpha diversity, patients were stratified at each time point into high versus low groups, dichotomized based on the median SDI. Antibiotic exposure was analyzed using two classifications: exposure before study inclusion (within 3 months), exposure during CRT, or no antibiotic exposure; and according to antibiotic spectrum [28] as narrow‐spectrum, broad‐spectrum, or no antibiotic exposure. Patients who had received antibiotics before CRT were excluded from the spectrum‐based analysis to permit longitudinal comparison across exposure groups. Beta diversity (between‐sample difference in overall gut bacterial composition) was evaluated using Bray–Curtis dissimilarity. Group differences were tested using multivariate permutational analysis of variance (PERMANOVA) adjusted for age, body mass index (BMI), PD‐L1 status, and dichotomized survival outcome groups (PFS/OS), and visualized by principal coordinate analysis (PCoA). For significant PERMANOVA findings, homogeneity of group dispersions was assessed using betadisper analysis. Differentially abundant taxa between survival groups at relevant time points were identified using Linear Discriminant Analysis (LDA) Effect Size (LEfSe). Taxa with LDA score ≥ 4.0 were considered of potential biological relevance and selected for exploratory reporting. All statistical analyses were performed in R (R version 4.5.1 (2025‐06‐13)). Packages used for statistical analyses and visualization included vegan (v2.7–1), phyloseq (v1.46.0), microbiomeMarker (v1.8.0), survival (v3.8–3), ggplot2 (v3.5.2), ggpubr (v0.6.1), dplyr (v1.1.4), and tidyr (v1.3.1). Two‐sided *P*‐values < 0.05 were considered statistically significant. No correction for multiple testing was performed; *P*‐values are nominal and interpreted descriptively.

### Ethics and regulatory approvals

This study was conducted as part of the DART trial in accordance with the Declaration of Helsinki [29], Good Clinical Practice guidelines, and applicable national regulations. The protocol was approved by the Regional Committee for Medical and Health Research Ethics of South‐East Norway (reference 48 655, November 28, 2019), and the Norwegian Medicines Agency. All participants provided written informed consent. The trial is registered at ClinicalTrials.gov (NCT04392505).

## Results

Gut microbiota data were collected from 62 patients at one or more time points (Norway 53, Finland 3, and Lithuania 6 patients). The median age was 69 years (36–85), and 58% were male. Histologic subtypes included adenocarcinoma in 43%, squamous cell carcinoma in 47%, and other histology in 10% of patients. Median BMI was 24.9 (17.6–46.9), and 12 patients had type 2 diabetes mellitus. At the time of data cutoff, the median follow‐up was 40.6 months (95% CI: 36.3–48.1). The median PFS was 19.0 months (95% CI 13.6–49.5), and the median OS was 48.2 months (95% CI 33.8–not reached). Additional baseline characteristics are summarized in Table 1. In total, 156 samples were collected; 39 patients provided samples at all three time points, and six patients contributed baseline samples only. After preprocessing and filtering, microbiota profiling provided data on 100 bacterial species.

**Table 1:** Patient characteristics.

Characteristic	Value
Age–years	
Median (range)	69 (36–85)
Sex–*n* (%)	
Male	36 (58%)
Female	26 (42%)
BMI median (range)	24.9 (17.6–46.9)
Performance status, ECOG	
ECOG 0	22 (35%)
ECOG 1	40 (65%)
Histology	
Adenocarcinoma	27 (43%)
Squamous cell carcinoma	29 (47%)
Other	6 (10%)
Disease stage	
IIB	5 (8%)
IIIA	25 (40%)
IIIB	24 (39%)
IIIC	7 (11%)
Unknown	1 (2%)
Radiotherapy Dose	
60–65 Gy	17 (27%)
66 Gy	43 (69%)
Missing	2 (3%)
Antibiotic treatment	
Before inclusion (0–3 months)	4 (6%)
During CRT	13 (21%)
Non	45 (73%)
Median PFS months (95% CI)	19.0 (13.6–49.5)
Median OS months (95% CI)	48.2 (33.8–not reached)

### Alpha diversity

**Fig. 1:**
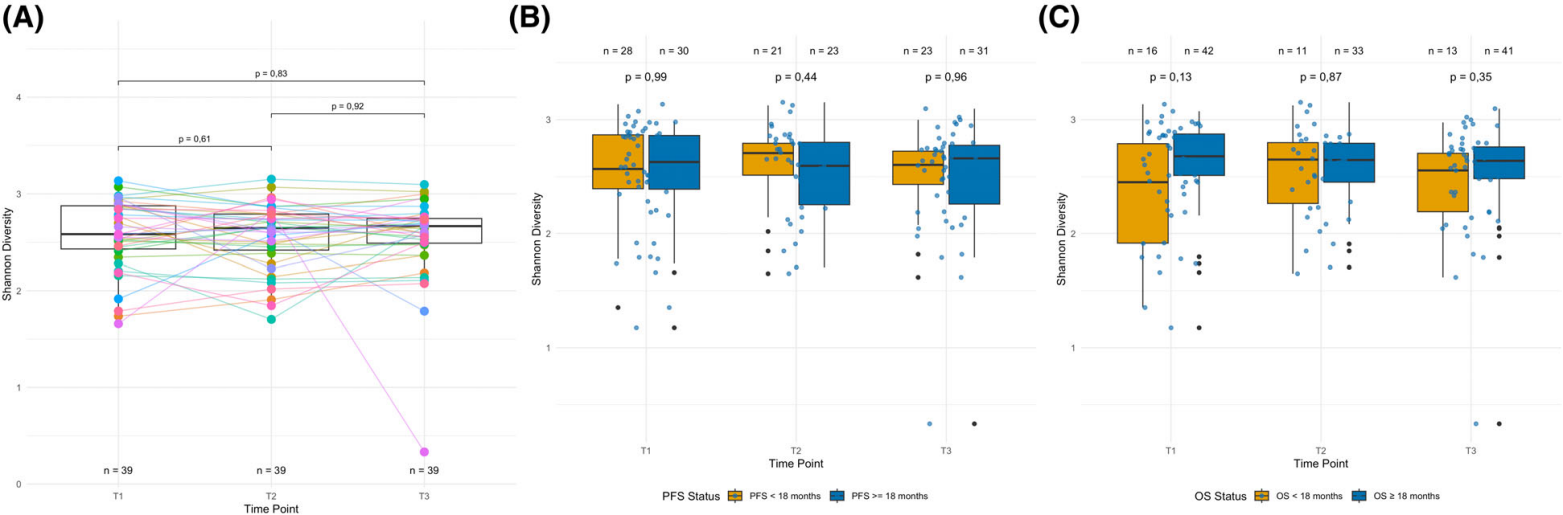
Alpha diversity according to progression‐free survival (PFS) and overall survival (OS). (A) Shannon Diversity Index across the three sampling time points: baseline (T1), postchemoradiotherapy (T2), and pre‐immunotherapy (T3), all *P* ≥ 0.61 (B) Alpha diversity by PFS ≥ 18 months (blue) or < 18 months (orange). (C) Alpha diversity by OS ≥ 18 months (blue) or < 18 months (orange), all *P* ≥ 0.13. Boxes show the median and the interquartile range (IQR: 25th–75th percentile); whiskers extend to the most extreme value within 1.5 × IQR of the box. Group comparisons were performed using the Wilcoxon rank‐sum test.

Within‐sample bacterial diversity remained stable across time points (median SDI 2.58, 2.65, and 2.67 at T1, T2, and T3, respectively; all *P* > 0.60) (Fig. 1A). Alpha diversity did not differ significantly between survival groups at any time point (PFS ≥ 18 vs < 18 months, all *P* ≥ 0.44; OS ≥ 18 vs < 18 months, all *P* ≥ 0.13) (Fig. 1B,C).

After stratification by PD‐L1 status (positive vs negative), no significant differences in alpha diversity were observed at any time point, although patients with PD‐L1‐positive tumors showed a trend toward higher baseline alpha diversity (T1, *P* = 0.079) (Fig. S2a). Within‐group trajectories were stable in both subgroups (all *P* ≥ 0.30) (Fig. S2b).

No significant differences in alpha diversity were observed between countries (Norway, Finland, and Lithuania) at any time point (all *P* ≥ 0.052), nor were significant temporal changes detected within countries (all *P* ≥ 0.62) (Fig. S3a,b), although the SDI was consistently higher in Norwegian samples across time points (Fig. S3b). Interpretation of country‐specific analyses is limited by the small sample sizes in the Finnish (*n* = 3) and Lithuanian (*n* = 6) cohorts.

**Fig. 2:**
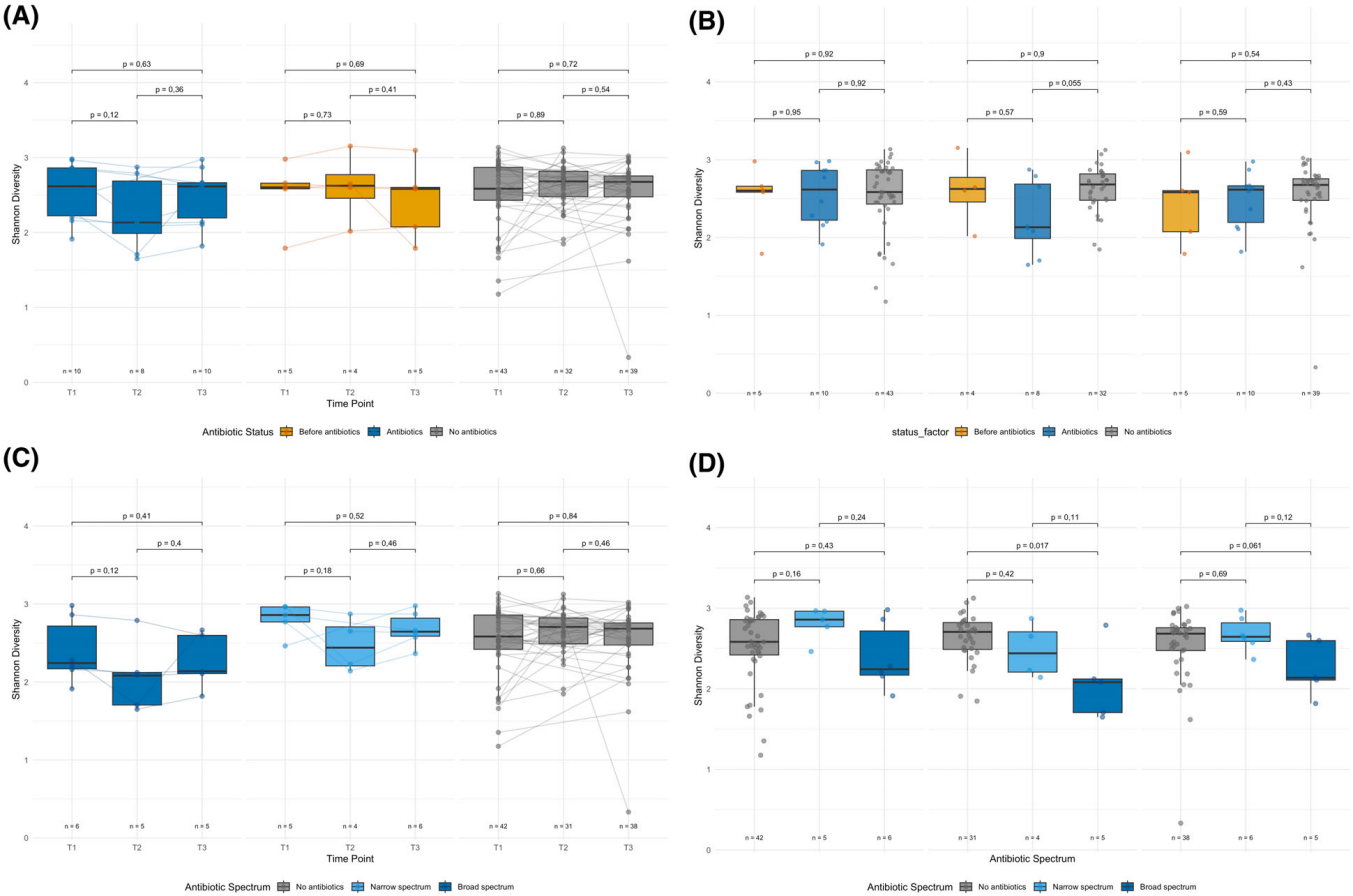
Alpha diversity according to antibiotic exposure. (A) Longitudinal changes in Shannon Diversity Index according to timing of antibiotic exposure: before chemoradiotherapy (CRT; orange), during CRT (blue), or no antibiotic exposure (gray), all *P* ≥ 0.12 (B) Shannon Diversity Index at T1, T2, and T3 according to timing of antibiotic exposure. A difference was observed at T2 between the antibiotic during CRT group (blue) and no antibiotic exposure group (gray), although this did not reach statistical significance; *P* = 0.055. All other *P* ≥ 0.43 (C) Longitudinal changes in Shannon Diversity Index according to antibiotic spectrum: narrow‐spectrum (light blue), broad‐spectrum (dark blue), or no antibiotic exposure (gray), all *P* ≥ 0.12 (D) Shannon Diversity Index at T1, T2, and T3 according to antibiotic spectrum. A significant difference was observed at T2 between patients receiving broad‐spectrum antibiotics (dark blue) and those without antibiotic exposure (gray); *P* = 0.017, all other *P* ≥ 0.061. Boxes show the median and the interquartile range (IQR: 25th–75th percentile), whiskers extend to the most extreme value within 1.5 × IQR of the box. Group comparisons were performed using the Wilcoxon rank‐sum test.

Alpha diversity was compared between patients who received antibiotics within 0–3 months prior to CRT (Before Antibiotics), during CRT (Antibiotics), and those without antibiotic exposure (No Antibiotics). From baseline (T1) to post‐CRT sampling (T2), alpha diversity decreased in patients exposed to antibiotics during CRT, although the change did not reach statistical significance (*P* = 0.12) (Fig. 2A). At T2, patients treated with antibiotics during CRT exhibited lower alpha diversity compared with patients without antibiotic exposure, with a trend toward statistical significance (*P* = 0.055) (Fig. 2B). To further evaluate the impact of antibiotic and antibiotic spectrum, patients with antibiotic exposure prior to CRT were excluded (*n* = 5), and treatment groups were categorized into narrow‐spectrum antibiotics, broad‐spectrum antibiotics, and no antibiotic exposure. Alpha diversity declined from T1 to T2 in both antibiotic‐treated groups, although neither reduction reached statistical significance (narrow‐spectrum *P* = 0.18; broad‐spectrum *P* = 0.12). In contrast, alpha diversity remained stable in the nonantibiotic group (*P* = 0.66) (Fig. 2C). At T2, alpha diversity was significantly lower in the broad‐spectrum antibiotic group than in the nonantibiotic group (*P* = 0.017) (Fig. 2D). Although the remaining between‐group comparisons were not statistically significant, alpha diversity showed a stepwise decline across treatment groups at T2, decreasing from no antibiotic treatment to narrow‐spectrum and broad‐spectrum antibiotic exposure (no antibiotics vs narrow‐spectrum: *P* = 0.42; narrow‐spectrum vs broad‐spectrum: *P* = 0.11) (Fig. 2D).

Kaplan–Meier analyses showed no significant association between SDI above or below the median and PFS or OS at any time point (PFS *P* = 0.80, 0.14, and 0.96; OS *P* = 0.32, 0.17, and 0.40) (Fig. S4a–f). Stratification by PD‐L1 status similarly revealed no significant associations between alpha diversity and either PFS or OS at any time point (all *P* ≥ 0.12) (Fig. S5a–f). No significant differences were found between the countries regarding PFS or OS (*P* = 0.16 and *P* = 0.25, respectively), remembering interpretation of country‐specific analyses is limited by the small sample sizes in the Finnish (*n* = 3) and Lithuanian (*n* = 6) cohorts (Fig. S6a,b). No significant differences in PFS or OS were observed by antibiotic exposure (exposure before CRT, during CRT, or no antibiotic exposure) or antibiotic spectrum status (all *P* ≥ 0.44) (Fig. S7a–d). As expected, PD‐L1‐positive status was associated with significantly longer PFS and OS (*P* = 0.0073 and *P* = 0.039, respectively) (Fig. S8a,b).

Among the 39 patients with complete longitudinal data, k‐means clustering identified two primary alpha diversity trajectories. Cluster A (*n* = 27) demonstrated a modest decline from T1 to T3 (mean SDI delta: −0.104), while Cluster B (*n* = 11) showed an initial dip at T2, followed by recovery (mean SDI delta T: +0.142) (Fig. S9). Antibiotic exposure during CRT was more prevalent in Cluster B (40%) than in Cluster A (12%), consistent with transient antibiotic‐associated changes in diversity.

### Beta diversity

Between‐sample compositional differences (beta diversity) were largely explained by patient identity (77%; *R* ^2^ = 0.77, *P* = 0.001), whereas time (treatment) explained only 1% of the variation (*R* ^2^ = 0.01, *P* = 0.001) (Fig. S10).

**Fig. 3:**
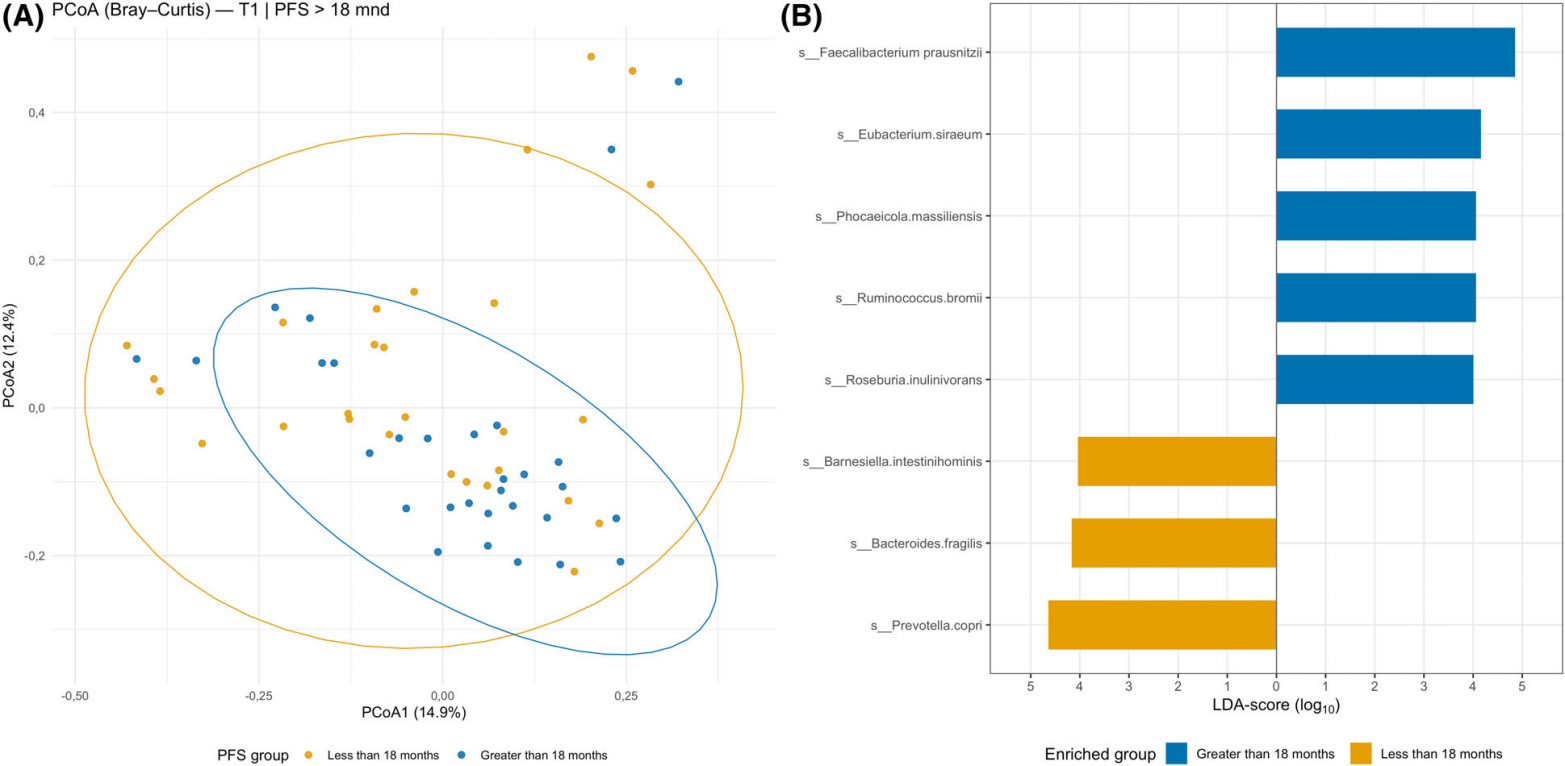
Baseline beta diversity and differential abundance according to progression‐free survival (PFS). (A) Principal coordinates analysis of beta diversity at baseline (T1) in patients with PFS ≥ 18 months (blue) and < 18 months (orange) (PERMANOVA, *R* ^2^ = 0.028, *P* = 0.045). (B) Exploratory differential abundance analysis at baseline identified three taxa enriched in patients with PFS < 18 months (orange) and five taxa enriched in patients with PFS ≥ 18 months (blue). Abbreviation: LDA, linear discriminant analysis; PCoA, principal coordinates analysis.

At baseline (T1), PERMANOVA adjusted for age, PD‐L1 expression, and BMI revealed a modest but statistically significant difference in gut microbial beta diversity between PFS groups (*R* ^2^ = 0.028, *P* = 0.045), with no evidence of unequal multivariate dispersion (betadisper: *F* = 1.534, *P* = 0.221) (Fig. 3A). Intra‐individual microbial stability was assessed using Bray–Curtis dissimilarity between baseline and follow‐up samples. No significant differences in within‐subject microbial variability were observed between PFS groups at either T1 → T2 or T1 → T3 (*P* = 0.887 and *P* = 0.869, respectively) (Fig. S11). Due to the limited sample size in two of the country cohorts (*n* = 3 and *n* = 6), adjusted PERMANOVA models could not be reliably estimated and were therefore not further explored. Adjusting for antibiotic use, categorized as before treatment, during treatment, or no exposure, was not significantly associated with microbial community composition. Stratification by antibiotic spectrum revealed no significant difference in microbial community composition prior to antibiotic treatment exposure at T1, but a significant difference was observed at T2 (*R* ^2^ = 0.075, *P* = 0.026) and T3 (*R* ^2^ = 0.062, *P* = 0.036), without heterogeneous dispersion (T2: *F* = 0.826, *P* = 0.446; T3: *F* = 0.707, *P* = 0.498) (Fig. S12a–c). The association between PFS groups and microbial community composition remained significant across adjusted analyses.

### Taxa associated with PFS groups

The significant baseline beta diversity difference between PFS groups prompted exploratory analyses. Using an LDA threshold of ≥ 4, LEfSe identified three taxa nominally enriched in patients with shorter PFS: *Prevotella copri*, *Bacteroides fragilis*, and *Barnesiella intestinihominis*. Five taxa were nominally enriched in patients with longer PFS: *Faecalibacterium prausnitzii*, *Ruminococcus bromii*, *Eubacterium siraeum*, *Phocaeicola massiliensis*, and *Roseburia inulinivorans* (Fig. 3B).

## Discussion

In this prospective cohort of patients with locally advanced NSCLC receiving concurrent CRT, overall gut microbiota within‐sample diversity (alpha diversity) remained stable from baseline through CRT completion and until immunotherapy initiation four weeks later. Qiu et al. similarly found stable alpha diversity in patients with lung cancer undergoing CRT [27]. Between‐sample differences in overall composition (beta diversity) were mainly explained by interpatient variation (77%) rather than treatment over time (1%). Baseline beta diversity differed modestly but significantly between PFS groups. Importantly, as with alpha diversity, beta diversity changed little from baseline through subsequent time points, suggesting that baseline microbial community structure may serve as a stable marker with potential predictive relevance.

Within‐sample diversity (alpha diversity) did not differ significantly between survival groups at any time points. The literature regarding alpha diversity and survival outcomes remains inconsistent. No association was reported in breast cancer and metastatic colorectal cancer receiving chemotherapy [30, 31], whereas higher alpha diversity during CRT correlated with longer PFS in lung cancer [27]. In platinum‐resistant ovarian cancer, declining alpha diversity during treatment was associated with poor outcomes [32], while higher alpha diversity was reported in chemotherapy‐resistant disease using a machine learning approach [33]. These divergent findings across cancer types, treatments, and analytical approaches underscore the need for standardized, adequately powered studies to clarify the role of alpha diversity as a biomarker for various clinical treatments.

Antibiotic exposure, particularly broad‐spectrum agents, was associated with reduced alpha diversity and differences in beta diversity, consistent with established antibiotic effects on the microbiota [34, 35]. However, neither antibiotic exposure nor antibiotic spectrum was associated with PFS or OS, possibly reflecting the transient nature of the perturbations observed. While our findings contrast with studies in patients with NSCLC reporting inferior survival among antibiotic‐exposed patients [36, 37], differences in patient population, treatment modalities, and study size may account for these discrepancies.

As all patients had responded to CRT and initiated immunotherapy, the analyses were not designed to assess microbial signatures of CRT response alone. The relative stability of microbiota composition during CRT, combined with significant baseline beta diversity differences by PFS, motivated exploratory taxa‐level analysis of baseline microbial composition. Eight bacterial taxa differed nominally between patients with PFS shorter versus longer than 18 months (Fig. 3B). Among those enriched in the short PFS‐group, *Prevotella copri* has been associated with inferior PFS in immunotherapy‐treated NSCLC in a metagenomic machine learning study [38], consistent with our findings, though enrichment in immunotherapy responders has been reported in the CheckMate 078 and 870 NSCLC cohorts [39]. A systematic review similarly found *P. copri* more frequently associated with responders, although results across studies were inconsistent [40]. These discrepancies may reflect context‐dependent effects related to host metabolism, diet, immune status, and strain‐level heterogeneity, as different *P. copri* strains exhibit different metabolic and immunomodulatory properties [41, 42]. *Bacteroides fragilis*, a bacterium with both pro‐and anti‐inflammatory properties depending on the underlying strain and environmental context [43], has mainly been investigated in colorectal cancer. A systematic review of rectal cancer reported increased abundance of *B. fragilis* among nonresponders to neoadjuvant treatment [44], whereas a study of advanced solid tumors receiving combined immunotherapy and chemotherapy found enrichment in responders [45]. In our cohort, *B. fragilis* was enriched in patients with shorter PFS. *Faecalibacterium prausnitzii*, butyrate‐producing bacterium with anti‐inflammatory properties [46], consistently linked to favorable immunotherapy [47] was likewise enriched in patients with longer PFS in our cohort. Similarly, *Ruminococcus bromii*, a key species in resistant starch degradation and anti‐inflammatory properties, has been proposed to support improved immunotherapy response [48, 49]. The remaining taxa identified at baseline are less consistently characterized as beneficial or nonbeneficial in the current literature, and several remain sparsely investigated [27, 50, 51, 52, 53, 54, 55, 56]. Collectively, these findings illustrate that while numerous candidate taxa have been proposed as biomarkers, results across studies remain inconsistent, likely reflecting methodological heterogeneity, variation in cancer type, treatment modality, taxonomic depth, and statistical approaches. Larger harmonized studies are needed to define reproducible, clinically meaningful microbial signatures.

This exploratory study aimed to identify potential microbial associations and possible candidate biomarkers in NSCLC patients undergoing CRT. Although our cohort of 62 patients is comparable to similar studies, the sample size remains limited, and incomplete sampling across time points further reduces statistical power. The marked imbalance in sample size across countries, particularly the two small cohorts, limited the assessment of geographic effects on microbiota composition, which has been shown to vary across populations and regions [57]. In addition, dietary data were not available, limiting the evaluation of an important determinant of gut microbial variation [58]. Microbial profiling was restricted to the predefined 108 taxa included in the PMP panel, preventing detection of bacteria outside this set. In addition, the lack of methodological harmonization across microbiome studies further complicates cross‐study comparisons. Despite these limitations, our findings provide insights and contribute to the growing evidence linking gut microbiota to lung cancer and its treatment.

## Conclusion

In our patient cohort with locally advanced, unresectable NSCLC receiving concurrent CRT prior to immunotherapy, the within‐sample diversity of gut microbiota (alpha diversity) remained stable across the CRT period. Antibiotics exposure during CRT induced a transient reduction in alpha diversity that recovered within weeks and did not translate into differences in PFS or OS.

A small but significant difference in between‐sample overall gut bacterial composition (beta diversity) was observed at baseline between patients with long and short PFS (≥ 18 vs < 18 months). These findings suggest that baseline gut microbiota composition may have potential relevance, whereas CRT itself does not appear to induce major microbiome shifts that influence outcome. If confirmed, this would support the use of baseline stool samples as a practical and resource‐efficient sampling strategy in future studies.

Larger prospective studies are needed to validate these findings and further clarify the gut microbiota's role in NSCLC treatment.

## Conflict of interest

Hanne Marte Nymoen: Honoraria: AstraZeneca. Henrik Horndalsveen: Advisory board: Johnson & Johnson, Honoraria; AstraZeneca, Pfizer, Roche. Bjørn Henning Grønberg: Advisory boards: Janssen, MSD, Johnson & Johnson, Accord, AstraZeneca, Pharmacosmos, Immedica, BMS, Merck. Honoraria: MSD, Pfizer, AstraZeneca, Johnson & Johnson, Gilead, BMS, Eli Lilly, Accord, Immedica, Pharmacosmos, Roche. Trial steering committee: Eli Lilly. Research funding: Roche, AstraZeneca. Tarje Halvorsen: Advisory board: AstraZeneca, Sanofi, Immedica, MSD. Honoraria: AstraZeneca, Takeda, MSD, Pfizer, Johnson & Johnson. Research funding: Roche, AstraZeneca. Tesfaye Madebo: Advisory board: Johnson and Johnson. Honoraria: AstraZeneca, GlaxoSmithKline, Takeda AS, Merck Sharp & Dome. Jussi Koivunen: Honoraria: Roche, AstraZeneca, Johnson and Johnson, Bristol‐Myers Squibb, Merck Sharp & Dome, Amgen, Merck KGaA, Novartis, Sanofi, and Pfizer. Research Funding: Institutional grants from AstraZeneca and Roche outside of current study. Lecturing: Siemens Healthineers. Employment: Former employee of Faron Pharmaceuticals. Kersti Oselin: Advisory board: Merck Sharp & Dome, AstraZeneca, Roche. Research Funding: Optellum. Marianne Aanerud: Honoraria: Bristol‐Myers Squibb, AstraZeneca. Jarkko Ahvonen: Advisory board: AstraZeneca. Maria Silvoniemi: Advisory board: AstraZeneca, Merck Sharp & Dome, Johnson and Johnson, Bristol‐Myers Squibb, Pfizer, Roche. Honoraria: AstraZeneca, Merck Sharp & Dohme, Johnson and Johnson, Bristol‐Myers Squibb, Pfizer, Roche, Boehringer‐Ingelheim. Åsa Kristina Öjlert: Advisory board: Sanofi. Saima Jamil Farooqi: Honoraria: Bristol‐Myers Squibb, BeOne Medicines. Åslaug Helland: Research Funding: Roche, AstraZeneca, Novartis, Incyte, Eli Lilly, Bristol‐Myers Squibb, Ultimovacs, Merck, GlaxoSmithKline, Illumina, Nanopore, Johnson and Johnson. Advisory boards and Honoraria: ABBVIE, Takeda, AstraZeneca, Roche, Pfizer, Janssen, EliLilly, Bristol‐Myers Squibb, PierreFabre, Bayer, Merck Sharp & Dome, Novartis, Merck, Sanofi, Medicover. All funds go to Oslo University Hospital. Vilde Drageset Haakensen: Advisory board: AstraZeneca, Merck Sharp & Dohme, Johnson & Johnson, Novartis, Bristol‐Myers Squibb. Honoraria: AstraZeneca, Merck Sharp & Dome, Johnson & Johnson, Novartis, Bristol‐Myers Squibb, Pfizer, Takeda. The remaining authors declare no competing interests.

## Author contributions

ÅH and VDH were involved in conceptualization. HMN and ZZ were involved in data analysis and interpretation. ÅH was involved in funding acquisition. HH, MMB, BHG, TH, HMN, TM, MA, JK, KO, SC, NH, JA, MS, ÅKÖ, SJF, and VDH were involved in patient inclusion. TD was involved in biobanking. HMN was involved in original drafted manuscript. All authors were involved in reviewing and editing manuscript.

## Disclosure

The authors declare that generative AI was not used in the design, data interpretation, or scientific conclusions of this manuscript. AI tools were used for language refinement and assistance with coding. All content was reviewed and approved by the authors, who take full responsibility for the manuscript.

## Supporting information


**Table S1.** Inclusion and exclusion criteria.
**Fig. S1.** Treatment schedule and stool sample time points. Stool samples were collected at three predefined time points: T1, prior to chemoradiation therapy (CRT); T2, at completion of CRT; and T3, prior to initiation of durvalumab treatment, approximately four weeks after completion of CRT. Created with BioRender.com.
**Fig. S2.** Alpha diversity by PD‐L1 status. (a) Alpha diversity by Shannon Diversity Index (SDI) across the time points: baseline (T1), postchemoradiotherapy (T2), and pre‐immunotherapy stratified by PD‐L1 negative (orange) or PD‐L1 positive (blue) tumors. All *P* ≥ 0.079, boxes show the median and interquartile range (IQR: 25th–75th percentile), whiskers extend to the most extreme data point within 1.5 × IQR of the box. (b) Longitudinal changes in SDI according to PD‐L1 status, all *P* ≥ 0.3. Group comparisons were performed using the Wilcoxon rank‐sum test.
**Fig. S3.** Alpha diversity by country. (a) Alpha diversity by Shannon Diversity Index (SDI) across the time points: baseline (T1), postchemoradiotherapy (T2), and pre‐immunotherapy stratified by country: Finland (orange), Lithuania (green), Norway (blue). All *P* ≥ 0.052, boxes show the median and interquartile range (IQR: 25th–75th percentile), whiskers extend to the most extreme data point within 1.5 × IQR of the box. (b) Longitudinal changes in SDI by country, all *P* ≥ 0.6. Group comparisons were performed using the Wilcoxon rank‐sum test.
**Fig. S4.** Survival outcome stratified by high versus low alpha diversity. No significant differences were found in progression‐free survival (a–c) or overall survival (d–f) by high vs low alpha diversity (dichotomized at the median Shannon Diversity Index) at any of the three time points; T1, T2, T3. Composite figure of Kaplan–Meier survival curves, group differences within each panel were tested using the log‐rank test, all *P* ≥ 0.14.
**Fig. S5.** Survival outcome in PD‐L1 positive and PD‐L1 negative tumors stratified by high versus low alpha diversity. No significant differences were found in PD‐L1 positive tumors (a–c) or PD‐L1 negative tumors (d–f) by high vs low alpha diversity (dichotomized at the median Shannon Diversity Index) at any of the three time points; T1, T2, T3. Composite figure of Kaplan–Meier survival curves, group differences within each panel were tested using the log‐rank test, all *P* ≥ 0.12.
**Fig. S6.** Survival outcome by country. No significant progression‐free survival (a) or overall survival (b) differences were detected between countries: Finland (yellow), Lithuania (green), Norway (blue). Composite figure of Kaplan–Meier survival curves, group differences within each panel were tested using the overall log‐rank test, all *P* ≥ 0.12.
**Fig. S7.** Survival outcome by antibiotic treatment exposure. No significant progression‐free survival (PFS) (a) or overall survival (OS) (b) differences were detected between antibiotic exposure status stratified by exposure before chemoradiation therapy (CRT) (blue), exposure during CRT (yellow), or no antibiotic exposure (gray). No significant PFS (c) or OS (d) differences were detected between antibiotic exposure status stratified by antibiotic spectrum; narrow‐spectrum (blue), broad‐spectrum (red), or no antibiotic exposure (gray). Composite figure of Kaplan–Meier survival curves, group differences within each panel were tested using the overall log‐rank test, all *P* ≥ 0.44.
**Fig. S8.** Survival outcome by PD‐L1 status. Significant differences in progression‐free survival (*P* = 0.0073) (a) and overall survival (*P* = 0.039) (b) were found when stratifying according to PD‐L1 status. PD‐L1 negative (blue), PD‐L1 positive (yellow). Composite figure of Kaplan–Meier survival curves, group differences within each panel were tested using the log‐rank test.
**Fig. S9.** Shannon diversity Trajectories by KML clustering. Longitudinal Shannon diversity trajectories grouped by K‐means for longitudinal data cluster (KML) of patients with samples at all three time points identified two distinct trajectories. Cluster A (*n* = 27) showed a modest decline in Shannon Diversity Index (SDI) from T1 to T3 (mean SDI: 2.84, 2.77, 2.71; ΔT1–T3 = −0.104). Cluster B (*n* = 11) exhibited an overall increase in SDI (mean SDI: 2.21, 2.04, 2.36; ΔT1–T3 = 0.142), with a transient decrease at T2. Antibiotic exposure during chemoradiation therapy was more frequent in Cluster B (40%) than in Cluster A (12%), consistent with transient antibiotic‐associated perturbations in diversity. No significant test is applied to this figure.
**Fig. S10.** Between‐sample microbial variation. Between‐sample variation (beta diversity) was largely explained by patient identity (77%; PERMANOVA, *R* ^2^ = 0.77, *P* = 0.001), whereas time (treatment) explained only 1% of the variation (PERMANOVA, *R* ^2^ = 0.01, *P* = 0.001; test of multivariate dispersion betadisper T1‐T3, *F* = 1.21, *P* = 0.301). Abbreviation: PCoA, Principal coordinates analysis.
**Fig. S11.** Within‐subject microbial variability by survival groups. Within‐subject microbial variability was assessed using Bray–Curtis dissimilarity between baseline (T1) and follow‐up samples (T2 and T3), stratified by progression‐free survival (PFS) group (≤ 18 vs > 18 months). No significant differences in intra‐individual microbial variability were observed between groups at either T1 → T2 (*P* = 0.89) or T1 → T3 (*P* = 0.87). Boxes show the median and interquartile range (IQR: 25th–75th percentile), whiskers extend to the most extreme data point within 1.5 × IQR of the box. Group comparisons were performed using the Wilcoxon rank‐sum test.
**Fig. S12.** Beta diversity by antibiotic treatment status. Principal coordinates analysis of beta diversity at T1 (a), T2 (b), and T3 (c) in patients with no antibiotic exposure (yellow), narrow‐spectrum antibiotic exposure (green), or broad‐spectrum antibiotic exposure (blue). Differences in community composition were observed at T2 (PERMANOVA, *R* ^2^ = 0.077, *P* = 0.026) and T3 (*R* ^2^ = 0.062, *P* = 0.036), but not at T1 (*P* = 0.421). Multivariate dispersion did not differ between groups at T2 (PERMDISP, *F* = 0.826, *P* = 0.446) or T3 (*F* = 0.707, *P* = 0.498). Abbreviation: PCoA, Principal coordinates analysis.

## Data Availability

The datasets generated and analyzed during the current study are not publicly available due to participant privacy and confidentiality considerations. Requests for access to de‐identified data may be directed to the corresponding author, Vilde Drageset Haakensen (email: vdd@ous-hf.no), and will be considered in accordance with applicable ethical and institutional regulations. Summary‐level data supporting the findings of this study are provided within the article and its Supporting Information.
