# Supplementary material for: Gut microbiota alterations in patients with non‐small‐cell lung cancer undergoing chemoradiotherapy
Source: Mol Oncol. 2026 Sep 7:10.1002/1878-0261.70326. Online ahead of print. doi: 10.1002/1878-0261.70326 (PMC13548291; doi:10.1002/1878-0261.70326)
Supplement: Supplementary file 1 [file MOL2-9999-0-s001.docx]

**Supplementary files for “Gut microbiota changes in patients with non-small cell lung cancer undergoing chemoradiotherapy”.**

**Supplementary Table 1. Inclusion and exclusion criteria.**

**Inclusion criteria**

For inclusion in the study patients must fulfill all of the following criteria:

1. Locally-advanced, unresectable, stage III Non-small cell lung cancer (NSCLC) (including PET-CT and MRI-brain in the diagnostic work-up).

2. Capable of giving signed informed consent which includes compliance with the requirements and restrictions listed in the informed consent form (ICF) and in the protocol. Written informed consent and any locally required authorization (European Union [EU] Data Privacy Directive in the EU) obtained from the patient/legal representative prior to performing any protocol-related procedures, including screening evaluations.

3. Diagnostic biopsy with PD-L1 <1% in 50 patients PD-L1 ≥1% in 50 patients

4. Adequate core or excisional biopsy for tumor assessment

5. Age > 18 years at time of study entry

6. Eastern Cooperative Oncology Group (ECOG) performance status of 0 or 1

7. Life expectancy of at least 12 weeks

8. Body weight >30 kg

9. Adequate normal organ and marrow function as defined below:

− Haemoglobin ≥9.0 g/dL

− Absolute neutrophil count (ANC) 1.5 x (> 1500 per mm3)

− Platelet count ≥100 x 109/L (>75,000 per mm3)

− Serum bilirubin ≤1.5 x institutional upper limit of normal (ULN).

− AST (SGOT)/ALT (SGPT) ≤2.5 x institutional upper limit of normal unless liver	metastases are present, in which case it must be ≤5x ULN

− Measured creatinine clearance (CL) >40 mL/min or Calculated creatinine CL>40 mL/min by the Cockcroft- Gault formula (Cockcroft and Gault 1976) or by 24-hour urine collection for determination of creatinine clearance:

Males: Creatinine CL (mL/min) =	Weight (kg) x (140 – Age)/	72 x serum creatinine (mg/dL)

Females: Creatinine CL (mL/min) =	Weight (kg) x (140 – Age)/	x 0.85

72 x serum creatinine (mg/dL)

10. Evidence of post-menopausal status or negative urinary or serum pregnancy test for female pre-menopausal patients. Women will be considered post-menopausal if they have been amenorrheic for 12 months without an alternative medical cause.

11. Women of childbearing potential (WOCBP) should have a negative urine or serum pregnancy within 7 days prior to receiving the first dose of study medication. If the urine test is positive or cannot be confirmed as negative, a serum pregnancy test will be required. A woman is considered as WOCBP, i.e. fertile, following menarche and until becoming post-menopausal unless permanently sterile. Permanent sterilisation methods include hysterectomy, bilateral salpingectomy and bilateral oophorectomy.

12. WOCBP should use an adequate method to avoid pregnancy

13. Males who are sexually active with women of childbearing potential must agree to follow instructions for method(s) of contraception for a period of 90 days (duration of sperm turnover) / the time required for the investigational drug to undergo five half-lives

14. Patient is willing and able to comply with the protocol for the duration of the study including undergoing treatment and scheduled visits and examinations including follow up.

**Exclusion criteria**

Patients should not enter the study if any of the following exclusion criteria are fulfilled:

1. Non-small cell lung cancer disease suitable for curative surgery

2. Significant cardiac, pulmonary or other medical illness that would limit activity or survival

3. Concurrent enrolment in another clinical study, unless it is an observational (non-interventional) clinical study or during the follow-up period of an interventional study during the last 2 weeks.

4. Any concurrent chemotherapy, Investigational Product (IP), biologic, or hormonal therapy for cancer treatment. Concurrent use of hormonal therapy for non-cancer-related conditions (e.g., hormone replacement therapy) is acceptable.

5. Major surgical procedure (as defined by the Investigator) within 28 days prior to the first dose of IP. Note: Local surgery of isolated lesions for palliative intent is acceptable.

6. History of allogenic organ transplantation.

7. Active or prior documented autoimmune or inflammatory disorders (including inflammatory bowel disease [e.g.,colitis or Crohn's disease], diverticulitis [with the exception of diverticulosis], systemic lupus erythematosus, Sarcoidosis syndrome, or Wegener syndrome [granulomatosis with polyangiitis, Graves' disease, rheumatoid arthritis, hypophysitis, uveitis, etc]). The following are exceptions to this criterion:

a. Patients with vitiligo or alopecia

b. Patients with hypothyroidism (e.g., following Hashimoto syndrome) stable on hormone replacement

c. Any chronic skin condition that does not require systemic therapy

d. Patients without active disease in the last 5 years may be included but only after consultation with the study physician

e. Patients with celiac disease controlled by diet alone

8. Uncontrolled intercurrent illness, including but not limited to, ongoing or active infection, symptomatic congestive heart failure, uncontrolled hypertension, unstable angina pectoris, cardiac arrhythmia, interstitial lung disease, serious chronic gastrointestinal conditions associated with diarrhea, or psychiatric illness/social situations that would limit compliance with study requirement, substantially increase risk of incurring AEs or compromise the ability of the patient to give written informed consent

9. History of another primary malignancy except for

a. Malignancy treated with curative intent and with no known active disease ≥5 years before the first dose of IP and of low potential risk for recurrence

b. Adequately treated non-melanoma skin cancer or lentigo maligna without evidence of disease

c. Adequately treated carcinoma in situ without evidence of disease

10. History of active primary immunodeficiency or medical condition requiring high doses (>30 mg prednisolone daily) of systemic steroids or other forms of immunosuppressive therapy

11. Active infection including tuberculosis (clinical evaluation that includes clinical history, physical examination and radiographic findings, and TB testing in line with local practice), hepatitis B (known positive HBV surface antigen (HBsAg) result), and hepatitis C. Patients with a past or resolved HBV infection (defined as the presence of hepatitis B core antibody [anti-HBc] and absence of HBsAg) are eligible. Patients positive for

hepatitis C (HCV) antibody are eligible only if polymerase chain reaction is negative for HCV RNA.

12. Current or prior use of immunosuppressive medication within 14 days before the first dose of durvalumab. The following are exceptions to this criterion:

a. Intranasal, inhaled, topical steroids, or local steroid injections (e.g., intra articular injection)

b. Systemic corticosteroids at physiologic doses not to exceed 10 mg/day of prednisone or its equivalent

c. Steroids as premedication for hypersensitivity reactions (e.g., CT scan premedication)

13. Receipt of live attenuated vaccine within 30 days prior to the first dose of IP. Note: Patients, if enrolled, should not receive live vaccine whilst receiving IP and up to 30 days after the last dose of IP.

14. Female patients who are pregnant or breastfeeding or male or female patients of reproductive potential who are not willing to employ effective birth control from screening to 90 days after the last dose of durvalumab monotherapy.

15. Known allergy or hypersensitivity to any of the study drugs or any of the study drug excipients.

16. Prior randomisation or treatment in a previous durvalumab clinical study regardless of treatment arm assignment.

17. Judgement by the investigator that the patient is unsuitable to participate in the study and the patient is unlikely to comply with study procedures, restrictions and requirements.

**Supplementary Figure 1.** **Treatment schedule and stool sample timepoints.**

Stool samples were collected at three predefined timepoints: T1, prior to chemoradiation therapy (CRT); T2, at completion of CRT; and T3, prior to initiation of durvalumab treatment, approximately four weeks after completion of CRT. Created with BioRender.com.

**Supplementary Figure 2.** **Alpha diversity by PD-L1 status.**

**(a)** Alpha diversity by Shannon diversity index (SDI) across the time points: baseline (T1), post-chemoradiotherapy (T2), and pre-immunotherapy stratified by PD-L1 negative (orange) or PD-L1 positive (blue) tumors. All p ≥ 0.079, boxes show the median and interquartile range (IQR: 25th–75th percentile), whiskers extend to the most extreme data point within 1.5×IQR of the box. **(b)** Longitudinal changes in SDI according to PD-L1 status, all p ≥ 0.3. Group comparisons were performed using the Wilcoxon rank-sum test.

**Supplementary Figure 3. Alpha diversity by country.**

**(a)** Alpha diversity by Shannon diversity index (SDI) across the time points: baseline (T1), post-chemoradiotherapy (T2), and pre-immunotherapy stratified by country: Finland (orange), Lithuania (green), Norway (blue). All p ≥ 0.052, boxes show the median and interquartile range (IQR: 25th–75th percentile), whiskers extend to the most extreme data point within 1.5×IQR of the box. **(b)** Longitudinal changes in SDI by country, all p ≥ 0.6. Group comparisons were performed using the Wilcoxon rank-sum test.

**Supplementary Figure 4. Survival outcome stratified by high versus low alpha diversity.**

No significant differences were found in progression-free survival **(a,b,c)** or overall survival **(d,e,f)** by high vs low alpha diversity (dichotomized at the median Shannon Diversity Index) at any of the three timepoints; T1, T2, T3. Composite figure of Kaplan-Meier survival curves, group differences within each panel were tested using the log-rank test, all p ≥ 0.14.

**Supplementary Figure 5. Survival outcome in PD-L1 positive and PD-L1 negative tumors stratified by high versus low alpha diversity.**

No significant differences were found in PD-L1 positive tumors **(a,b,c)** or PD-L1 negative tumors **(d,e,f)** by high vs low alpha diversity (dichotomized at the median Shannon Diversity Index) at any of the three timepoints; T1, T2, T3. Composite figure of Kaplan-Meier survival curves, group differences within each panel were tested using the log-rank test, all p ≥ 0.12.

**Supplementary Figure 6. Survival outcome by country.**

No significant progression-free survival **(a)** or overall survival **(b)** differences were detected between countries: Finland (yellow), Lithuania (green), Norway (blue). Composite figure of Kaplan-Meier survival curves, group differences within each panel were tested using the overall log-rank test, all p ≥ 0.12.

**Supplementary Figure 7. Survival outcome by antibiotic treatment exposure.**

No significant progression-free survival (PFS) **(a)** or overall survival (OS) **(b)** differences were detected between antibiotic exposure status stratified by exposure before chemoradiation therapy (CRT) (blue), exposure during CRT (yellow), or no antibiotic exposure (grey). No significant PFS **(c)** or OS **(d)** differences were detected between antibiotic exposure status stratified by antibiotic spectrum; narrow-spectrum (blue), broad spectrum (red), or no antibiotic exposure (gray). Composite figure of Kaplan-Meier survival curves, group differences within each panel were tested using the overall log-rank test, all p ≥ 0.44.

**Supplementary Figure 8. Survival outcome by PD-L1 status.**

Significant differences in progression-free survival (p = 0.0073) **(a)** and overall survival (p = 0.039) **(b)** were found when stratifying according to PD-L1 status. PD-L1 negative (blue), PD-L1 positive (yellow). Composite figure of Kaplan-Meier survival curves, group differences within each panel were tested using the log-rank test.

**Supplementary Figure 9. Shannon diversity Trajectories by KML clustering.**

Longitudinal Shannon diversity trajectories grouped by K-means for longitudinal data cluster (KML) of patients with samples at all three time points identified two distinct trajectories. Cluster A (n = 27) showed a modest decline in Shannon diversity index (SDI) from T1 to T3 (mean SDI: 2.84, 2.77, 2.71; ΔT1–T3 = −0.104). Cluster B (n = 11) exhibited an overall increase in SDI (mean SDI: 2.21, 2.04, 2.36; ΔT1–T3 = 0.142), with a transient decrease at T2. Antibiotic exposure during chemoradiation therapy was more frequent in Cluster B (40%) than in Cluster A (12%), consistent with transient antibiotic-associated perturbations in diversity. No significant test is applied to this figure.

**Supplementary Figure 10. Between-sample microbial variation.**

Between-sample variation (beta-diversity) was largely explained by patient identity (77%; PERMANOVA, R² = 0.77, p = 0.001), whereas time (treatment) explained only 1% of the variation (PERMANOVA, R² = 0.01, p = 0.001; test of multivariate dispersion betadisper T1-T3, F = 1.21, p = 0.301). Abbreviation: PCoA, Principal coordinates analysis.

**Supplementary Figure 11. Within-subject microbial variability by survival groups.**

Within-subject microbial variability was assessed using Bray–Curtis dissimilarity between baseline (T1) and follow-up samples (T2 and T3), stratified by progression-free survival (PFS) group (≤18 vs >18 months). No significant differences in intra-individual microbial variability were observed between groups at either T1→T2 (p = 0.89) or T1→T3 (p = 0.87). Boxes show the median and interquartile range (IQR: 25th–75th percentile), whiskers extend to the most extreme data point within 1.5×IQR of the box. Group comparisons were performed using the Wilcoxon rank-sum test.

**Supplementary Figure 12. Beta diversity by antibiotic treatment status.**

Principal coordinates analysis of beta diversity at T1 **(a)**, T2 **(b)**, and T3 **(c)** in patients with no antibiotic exposure (yellow), narrow-spectrum antibiotic exposure (green), or broad-spectrum antibiotic exposure (blue). Differences in community composition were observed at T2 (PERMANOVA, R² = 0.077, p = 0.026) and T3 (R² = 0.062, p = 0.036), but not at T1 (p = 0.421). Multivariate dispersion did not differ between groups at T2 (PERMDISP, F = 0.826, p = 0.446) or T3 (F = 0.707, p = 0.498). Abbreviation: PCoA, Principal coordinates analysis.
